# Some bee-pollinated plants provide nutritionally incomplete pollen amino acid resources to their pollinators

**DOI:** 10.1371/journal.pone.0269992

**Published:** 2022-08-02

**Authors:** Léna Jeannerod, Archibald Carlier, Bertrand Schatz, Clothilde Daise, Aurore Richel, Yannick Agnan, Mathilde Baude, Anne-Laure Jacquemart

**Affiliations:** 1 Earth and Life Institute—Agronomy, UCLouvain, Louvain-la-Neuve, Belgium; 2 CEFE, CNRS, Université de Montpellier, EPHE, IRD, Montpellier, France; 3 Biomass and Green Technologies, University of Liege—Gembloux Agro-Bio Tech, Gembloux, Belgium; 4 Université d′ Orléans, USC INRAE 1328, Laboratoire de biologie des ligneux et des grands cultures (LBLGC), Orléans, France; 5 Sorbonne Université, UPEC, Université Paris Cité, CNRS, IRD, INRAE, Institut d’Ecologie et des Sciences de l’Environnement (iEES-Paris), Paris, France; Universidade Federal de Uberlandia - Campus Umuarama, BRAZIL

## Abstract

For pollinators such as bees, nectar mainly provides carbohydrates and pollen provides proteins, amino acids, and lipids to cover their nutritional needs. Here, to examine differences in pollinator resources, we compared the amino acid profiles and total amino acid contents of pollen from 32 common entomophilous plants in seven families. Our results showed that the amino acid profiles and contents in pollen samples differed according to the plant family and the chromatography method used, i.e., high-performance liquid chromatography (HPLC) *versus* ion exchange chromatography (IEX). Pollen from Boraginaceae species had the highest total amino acid contents (361.2–504 μg/mg) whereas pollen from the Malvaceae family had the lowest total amino acid contents (136–243.1 μg/mg). Calculating an amino acid score (AAS) that reflects pollen nutritional quality showed that slightly less than half of the species (19 out of 32) had the maximum nutritional score (AAS = 1) and offered high nutritional quality pollen amino acids for bee pollinators. Though they had high total amino acid contents, the amino acid composition of the studied Boraginaceae species and several members of the Fabaceae was not optimal, as their pollen was deficient in some essential amino acids, resulting in suboptimal amino acid scores (AAS < 0.7). Except for cysteine, the measured amino acid contents were higher using IEX chromatography than using HPLC. IEX chromatography is more robust and is to be preferred over HPLC in future amino acid analyses. Moreover, our observations show that some bee-pollinated species fail to provide complete amino acid resources for their pollinators. Although the implications for pollinator behavior remain to be studied, these deficiencies may force pollinators to forage from different species to obtain all nutritionial requirements.

## Introduction

The majority of flowering plants (87% of all angiosperms) depend on animals for pollination and successful reproduction [1]. This essential pollination ecosystem service plays a key role in biodiversity and food production [2, 3]. Domestic and wild bees (Anthophila, *e*.*g*., honeybees, bumblebees, and solitary bees) pollinate the majority of plants in temperate zones. Evidence for declining populations of insects [4, 5], and pollinators in particular [6, 7], has accumulated in recent decades. The loss of floral resources from habitat destruction and degradation is considered one of the main drivers of pollinator decline [8, 9], as documented in agricultural areas [10–12].

In terms of floral resources, most bees depend exclusively on pollen and nectar for nutrition at all stages of their life cycle [9, 13, 14], although some specialized bees use other resources such as floral oils, carrion, or resins [15]. Nectar provides the main source of carbohydrates [16, 17] and pollen is the main source of proteins and lipids [16, 18–22]. As bees cannot synthesize amino acids *de novo*, pollen is crucial for larval growth and development as well as for adult growth, immune competence, longevity, and reproduction [19, 22–25]. Moreover, the amino acid composition of a food source a key determinant for a pollinator’s health and is more crucial than the protein content. Ten amino acids (arginine, histidine, isoleucine, leucine, lysine, methionine, phenylalanine, threonine, tryptophan, and valine) are considered essential for honeybees according to de Groot [26]. Deficiency of an essential amino acid in an insect diet will negatively affect protein synthesis, and thus fitness [27]. The choice of floral resources differs among pollinator species according to their particular nutritional requirements [23, 28]. Differences in pollen amino acid composition affect flower visitor behaviour, influencing olfactory learning and memory in honeybees and bumblebees [29, 30]. The amino acid score (AAS) is defined as the proportion of the limiting (lowest content) essential amino acid in pollen divided by the standard accepted proportions of essential amino acids according to de Groot [26] that are considered optimal for protein utilization [27, 31]. This AAS can be used to assess the nutritional quality of pollen.

In addition to providing nutrition for essential pollinators, the amino acids in pollen have functions in the plant. Pollen is required to fertilize ovules, and therefore to produce seeds. Although most of the nutrients needed for fertilization are provided by the recipient maternal plant [32], pollen contains enough nutrients to initiate the fertilization process through pollen tube growth, with pollen nutrient content related to the style length and distance between the receptive stigmatic surface and the ovules [20, 32]. Therefore, we can assume that related plants will have pollen with similar nutrient contents due to common metabolic pathways and similar floral morphologies (stigma–ovule distances). So, related plant species likely require similar amount and ratios of nutrients for effective fertilization. Differences in pollen nutritional content could therefore be largely determined by phylogenetic relatedness. For example, previous studies found a phylogenetic signal for pollen crude proteins and protein/lipid ratios [18, 29], and for pollen sterols [19, 33], but they have not considered amino acids contents and profiles.

For pollinator conservation efforts, the knowledge of the nutritional value of floral resources, including their quantity and chemical composition is required [10, 16–19, 34–36]. Although numerous studies have analysed the chemical composition and nutritional value of pollen resources, their analytical methods often differed and resulted in inconsistencies between the studies [16, 18, 20–22, 37–39]. Extraction, detection, identification, and quantification methods before and during the chromatography process influence the individual amino acid concentration results [21]. For example, one study showed that cysteine and methionine concentrations in pollen differed according to the method used, with higher results with IEX chromatography than with standard HPLC [40]. In addition, chemical analysis requires a substantial amount of pollen (100–150 mg), and it is easier to collect pollen from pellets brought back to the hive by bees than directly from flowers. Therefore, most studies have analysed the chemical compositions of bee-collected pollen rather than pure hand-collected pollen from individual flowers [21, 33, 41]. However, the nutrient composition of bee-collected pollen differs from pure floral pollen because insects add nectar and saliva to aggregate the pollen grains into a pellet [21].

In this study, we analyzed the amino acid profiles of pure floral pollen from 32 flowering plant species using two different chromatography methods: HPLC and IEX. In addition, we analyzed only pollen collected directly from flowers. Our objectives were to investigate the differences in the composition and concentration of pollen amino acids among different plant species and to compare how the different analytical methods influence their quantitation. We analyzed the total amino acid content and the individual concentration of each amino acid as well as the AAS for each plant species.

## Materials and methods

### Collection of pollen samples

We collected pure floral pollen from 32 entomophilous plant species (S1 Table) in a minimum of three Belgian locations and populations (from the three main biogeographical regions): the central loamy region (Ottignies, Court-Saint-Etienne, Hannut, Mons, and Brussels), Ardenne (Vielsalm, La-Roche-en-Ardenne, and Lierneux), and the southern Lorraine region (Arlon, Attert, Fouches, and Messancy) [31]. Plants were identified following the flora “Flore écologique de Belgique” [42]. Pollen collection challenges were: (i) efficient harvest of a sufficient mass of pure floral pollen; (ii) proper and immediate storage of pollen to prevent nutrient degradation; and (iii) accurate pollen weighing [18]. Floral pollen collection differed according to the morphology of the flower and involved three different techniques, classified as (i) “fresh”, (ii) “anther”, and (iii) “tiny”. In the “fresh” collection technique, inflorescences were laid flat on paper and pollen was collected directly from dehiscent anthers (Asteraceae). For open flowers, we brushed anthers with synthetic fine paint brushes or we cut anthers with fine scissors (Malvaceae, Rosaceae). In the “anther” technique, to expose the anthers of flowers with tubular corolla, such as Lamiaceae, Boraginaceae, and Sapindaceae, or keel-shaped flowers (Fabaceae), the petals were removed or cut (using fine scissors or scalpels). For the “tiny” technique, used to collect pollen from very small flowers (<5 mm Rosaceae; *Filipendula ulmaria*), flowers were collected in their entirety and pollen was sorted by sieving after flower drying.

Once collected, the pollen was placed in paper envelopes or glass vials. To prevent nutrient degradation, all pollen was stored at –20°C until required for analysis (flowers or pollen were placed on ice in the field). As heating can alter the chemical composition, pollen was dried at 36°C for 24 hours. Then pollen was sieved to remove plant debris (25–50 μm mesh depending on the pollen grain diameter, Brass–Stainless, FullHeigh, Standard test sieves, USA). Sieved pollen grains were checked for purity under a light microscope (Nikon, Gx100-400). The pollen from tens to hundreds of flowers of each plant species was pooled for each location to provide sufficient pollen for analysis. All samples were freeze-dried and carefully homogenized before analyses and all analyses were performed.

### Determination of amino acid profiles

#### IEX chromatography method

Pollen from 26 species (S1 Table) was analysed using the method developed in [22]. The analysis (including the two hydrolyses) was replicated several times (n = 3–6) for better uniformity of results. One milliliter of hydrolysis solution (6 N HCl, 0.1% phenol, and 500 mM norleucine [used as an internal standard]) was added to 3–5 mg pollen (dried weight). Tubes were placed in liquid nitrogen for one minute to avoid methionine degradation. Hydrolysis was conducted for 24 hours at 110°C. The hydrolysate was dried by vacuum in a boiling bath at 100°C. Afterwards, 1 mL of sodium citrate buffer pH 2.2 was added to the tube. The sample solution was mixed and poured in a HPLC vial after filtration (0.2 μm). Total amino acids were measured separately with an IEX chromatography machine (Biochrom 20 coupled with amino acid analyser). Only tryptophan was omitted because (1) its isolation requires a separate alkaline hydrolysis from a second sample, and (2) tryptophan is rarely a limiting essential amino acid [43].

#### HPLC method

Pollen from 12 species (S1 Table) was analysed using a standard HPLC method. Aqueous solution (100 μL) of 25 μM norleucine (used as an internal standard) was added to 3 mg dried pollen. Amino acids were quantified using HPLC after acid hydrolysis (with or without prior oxidation) and derivatization using o-phthaldialdehyde (OPA) reagent in combination with (9-fluorenylmethyl)chloroformate (Fmoc-Cl) [44]. Three replicates were used; two for the procedure with acid hydrolysis only and one for the procedure with oxidation and acid hydrolysis.

For acid hydrolysis, samples 150 μL 6 M HCl containing 1% (w/v) phenol was added to the samples, which were incubated for 18 h at 110°C. For acid hydrolysis with prior oxidation, 50 μL performic acid reagent (10 mg phenol crystal in 10 mL deionised water with 1 mL of 33% H_2_O_2_ and 9 mL of 88% formic acid) was added to the samples. After addition of the reagent, tubes were incubated for 3 hours at 4°C, and 8 mg sodium metabisulfite was then added to decompose the performic acid. Tubes were then stirred for 1.5 seconds to liberate any SO_2_ produced.

A double derivatization process was performed in pre-columns using OPA reagent in combination with Fmoc-Cl. Samples were injected into a Zorbax Eclipse Plus column (Agilent; 3.5 μm particle size; 150 × 21 mm) maintained at 40°C. The mobile phase was composed of (A) 40 mM phosphate buffer pH 8.4 and (B) acetonitrile/methanol/water (45:45:10 v/v/v) at a flow rate of 0.42 mL/min (100% A–0% B 0.5 min; progressive increase from 0% to 57% B 0.5–25 min). OPA-derivatized and Fmoc-Cl-derivatized amino acids were detected at 340 nm and 266 nm excitation and 450 nm and 338 nm emission wavelengths, respectively.

### Statistical analysis

We compared the pollen amino acid composition of 32 species belonging to seven plant families (Asteraceae, Boraginaceae, Fabaceae, Lamiaceae, Malvaceae, Rosaceae, and Sapindaceae). Each family included 2 to 9 species. All analyses were performed in R 3.6.1.

#### Total amino acids

To compare the analytical methods used to determine the chemical composition of pollen species, we first tested the influence of the method on the total amino acids (the sum of the free amino acids and the protein-bound amino acids). We fitted a linear model on the total amino acid content of the 32 species according to the ‘method’ (the protocol performed to analyse the amino acid composition) and the ‘plant family’, with interaction effects between the two predictors.

We fitted a linear model on the total amino acid content for six plant species analysed with the two methods (*Aesculus hippocastanum*, *Borago officinalis*, *Cyanus segetum*, *Cytisus scoparius*, *Lamium album*, and *Pyrus communis*). For each model, residual normality and heteroscedasticity were visually checked using residual plots *versus* fitted values and QQ-plot, respectively, followed by a two-way ANOVA to determine the effects of variables.

#### Amino acid composition

The differences in amino acid composition for the 32 floral species according to the analytical method were visually assessed on a non-metric multidimensional scaling (nMDS) ordination using a Bray-Curtis similarity matrix, with two dimensions using the ‘*metaMDS*’ command and 50 iterations (R-package *vegan* [45]). To test differences in amino acid compositions, a perMANOVA was performed using the Bray–Curtis dissimilarity matrix and 999 permutations (*Adonis* command, R-package *vegan* [45]). Prior to the permutation analysis of variance, the multivariate homogeneity of within-group covariance matrices was checked using the *betadisper* function.

The contents of each amino acid were compared in a MANOVA using ‘method’ and ‘family’ as main effects (with interaction effects between the two predictors) and the contents of each amino acid as separate dependent variables.

#### Amino acid score

A global AAS was calculated for each species, with the proportion of the limiting (lowest content) essential amino acid in the protein of the pollen divided by the standard accepted proportions of essential amino acids according to de Groot [26]. We fitted a linear model on the AAS of the 32 species according to the ‘plant family’ and the ‘method’. Residual normality and heteroscedasticity were visually checked using the plot of residuals versus fitted values and QQ-plot respectively, prior to two-way ANOVA.

## Results

All the analysed plant species contained all the essential amino acids, as well as almost all the other amino acids, except for cysteine (S1 Table). The six amino acids found in the highest abundance regardless of the species were aspartic acid, glutamic acid, glycine, leucine, lysine, and proline (S1 Table).

### Total amino acids

**Fig 1:**
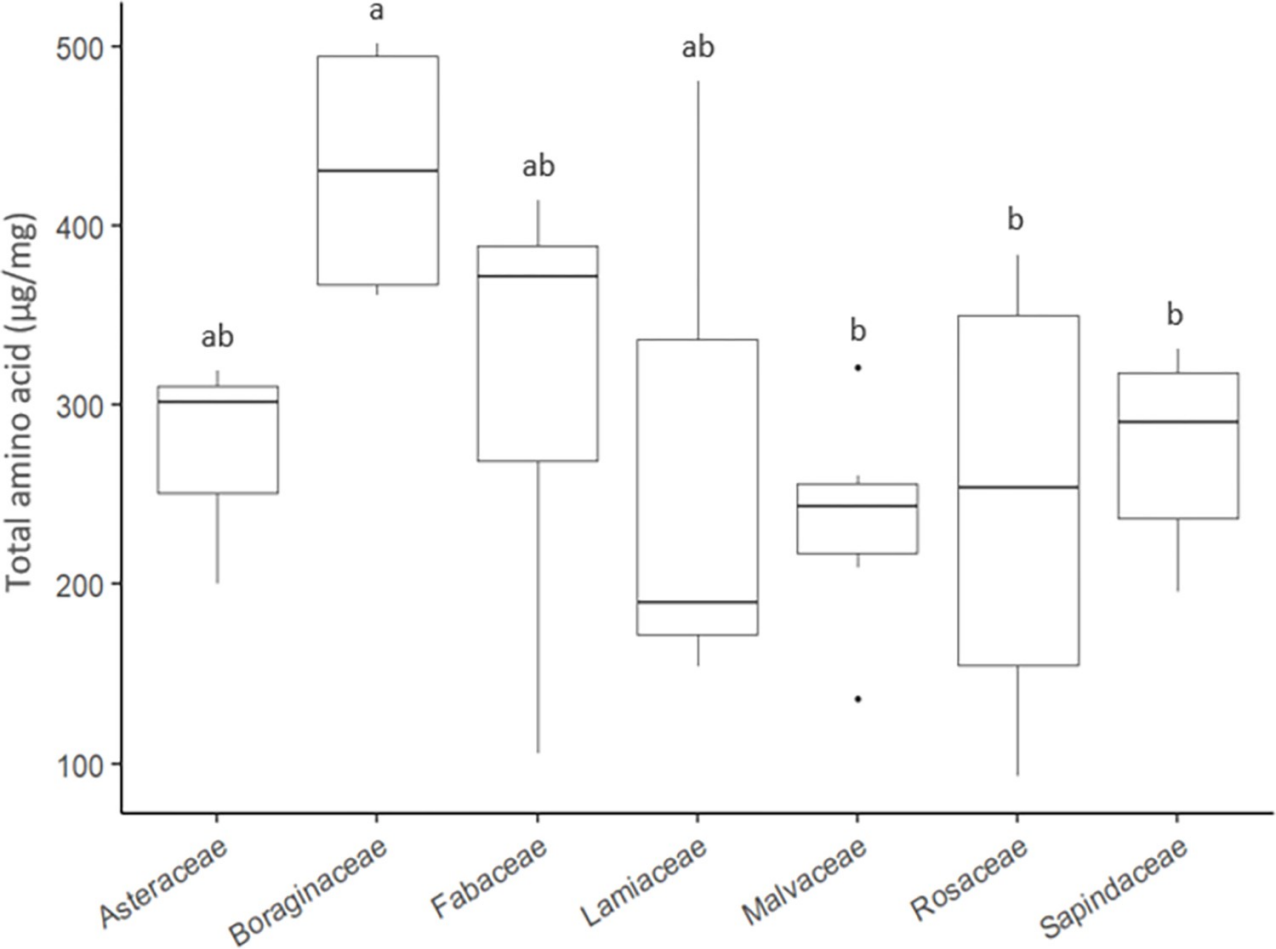
Boxplot of total amino acids per plant family.

For the 32 species analyzed, total amino acid contents differed according to the analytical method (*F* = 39.161 *d*.*f*. = 1, *p* < 0.001) and to the family (*F* = 4.733, *d*.*f*. = 6, *p* < 0.01). The interaction between method and family was not significant (*F* = 1.663, *d*.*f*. = 6, *p* > 0.05), indicating a similar effect of the analytical method regardless of the plant family. Pollen from Boraginaceae species contained the highest total amino acid contents (361.2–504 μg/mg) regardless of the method used, whereas pollen from the Malvaceae family contained the lowest total amino acid contents (136–243.1 μg/mg, Fig 1).

For the six species for which pollen was analysed with both methods (*Aesculus hippocastanum*, *Borago officinalis*, *Cyanus segetum*, *Cytisus scoparius*, *Lamium album*, and *Pyrus communis*), total amino acid contents were significantly higher with IEX chromatography compared with HPLC (*F* = 30.203, *d*.*f*. = 1, *p* < 0.01) whereas no differences were detected among the species (*F* = 2.635, *d*.*f*. = 5, *p* > 0.05).

#### Amino acid composition

**Fig 2:**
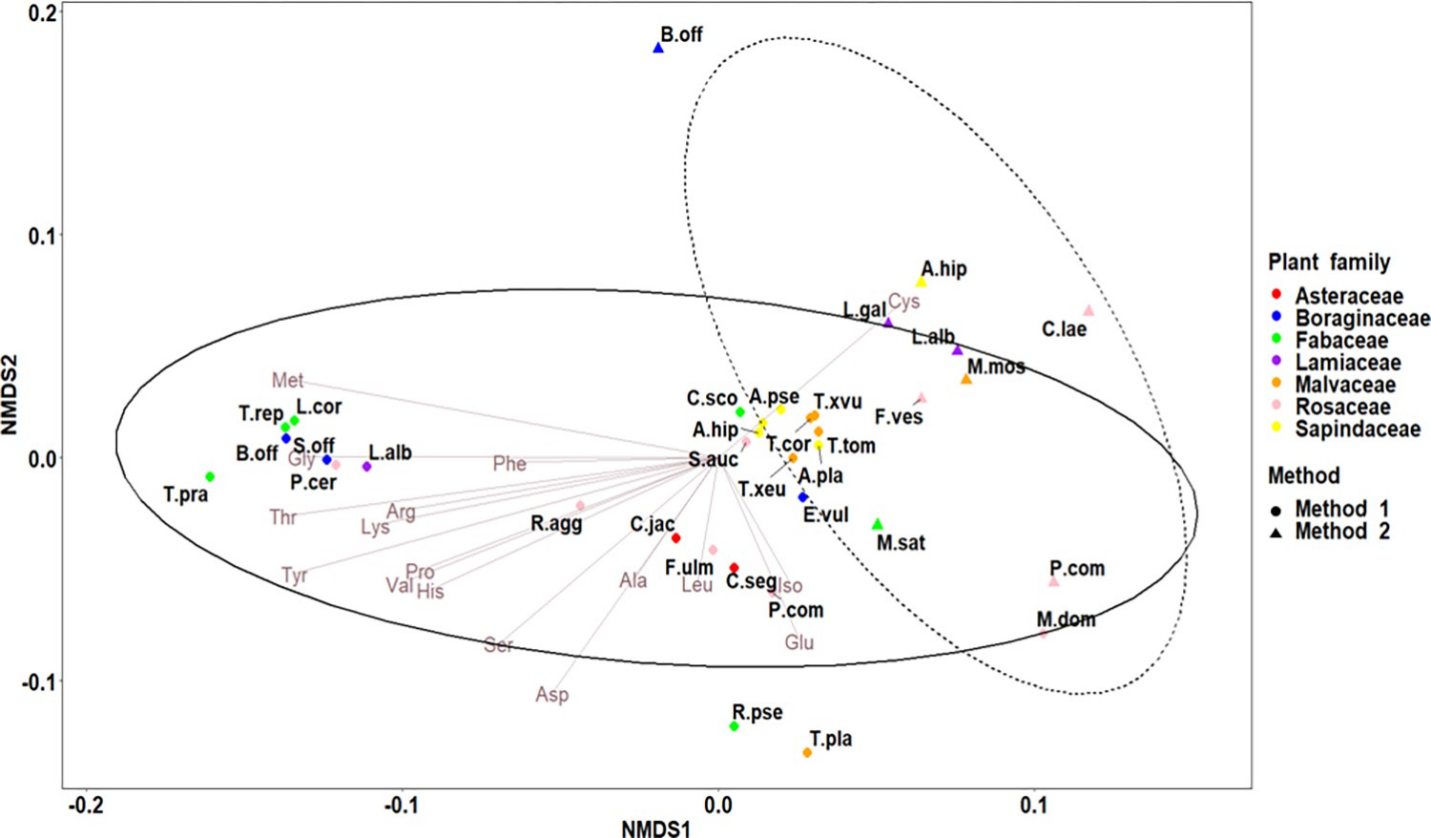
nMDS ordination plot based on Bray-Curtis distances calculated on amino acids contents (i.e., amounts in μg/mg of amino acids) from pure floral pollen for each plant species (stress = 0.09). Method 1: IEX chromatography method; Method 2: HPLC method. The two ellipses gather species according to the analytical method used with solid lines for the IEX chromatography method and dashed lines for the HPLC method. Families are indicated in different colours. Amino acids: Ala, alanine; arg, arginine; asp, aspartic acid; cys, cysteine; glu, glutamine; gly, glycine; his, histidine; ile, isoleucine; leu, leucine; lys, lysine; met, methionine; phe, phenylalanine; pro, proline; ser, serine; thr, threonine; tyr, tyrosine; val, valine. Species: A.pla, *Acer platanoides*; A.pse, *Acer pseudoplatanus*; A.car, *Aesculus carnea*; A.hip, *Aesculus hippocastanum*; B.off, *Borago officinali*s; C.jac, *Centaurea jacea*; C.lae, *Crataegus laevigata*; C.seg, *Cyanus segetum*; C.sco, *Cytisus scoparius*; E.vul, *Echium vulgare*; F.ulm, *Filipendula ulmaria*; F.ves, *Fragaria vesca*; L.alb, *Lamium album*; L.gal, *Lamium galeobdolon*; L.cor, *Lotus corniculatus*; M.mos, *Malva moschata*; M.dom, *Malus domestica*; P.avi, *Prunus avium*; P.cer, *Prunus cerasus*; P.com, *Pyrus communis*; R.pse, *Robinia pseudoacacia*; R.agg, *Rubus aggr*.; S.auc, *Sorbus aucuparia*; S.off, *Symphytum officinale*; T.cor, *Tilia cordata*; T.pla, *Tilia platyphyllos*; T.tom, *Tilia tomentosa*; T.xeu, *Tilia x euchlora*; T.xvu, *Tilia x vulgaris*; T.pra, *Trifolium pratense*; T.rep, *Trifolium repens*.

We also detected differences in amino acid composition between the two methods (PerMANOVA: *F* = 19.878; *d*.*f*. = 1; *p* < 0.001; Fig 2).

**Fig 3:**
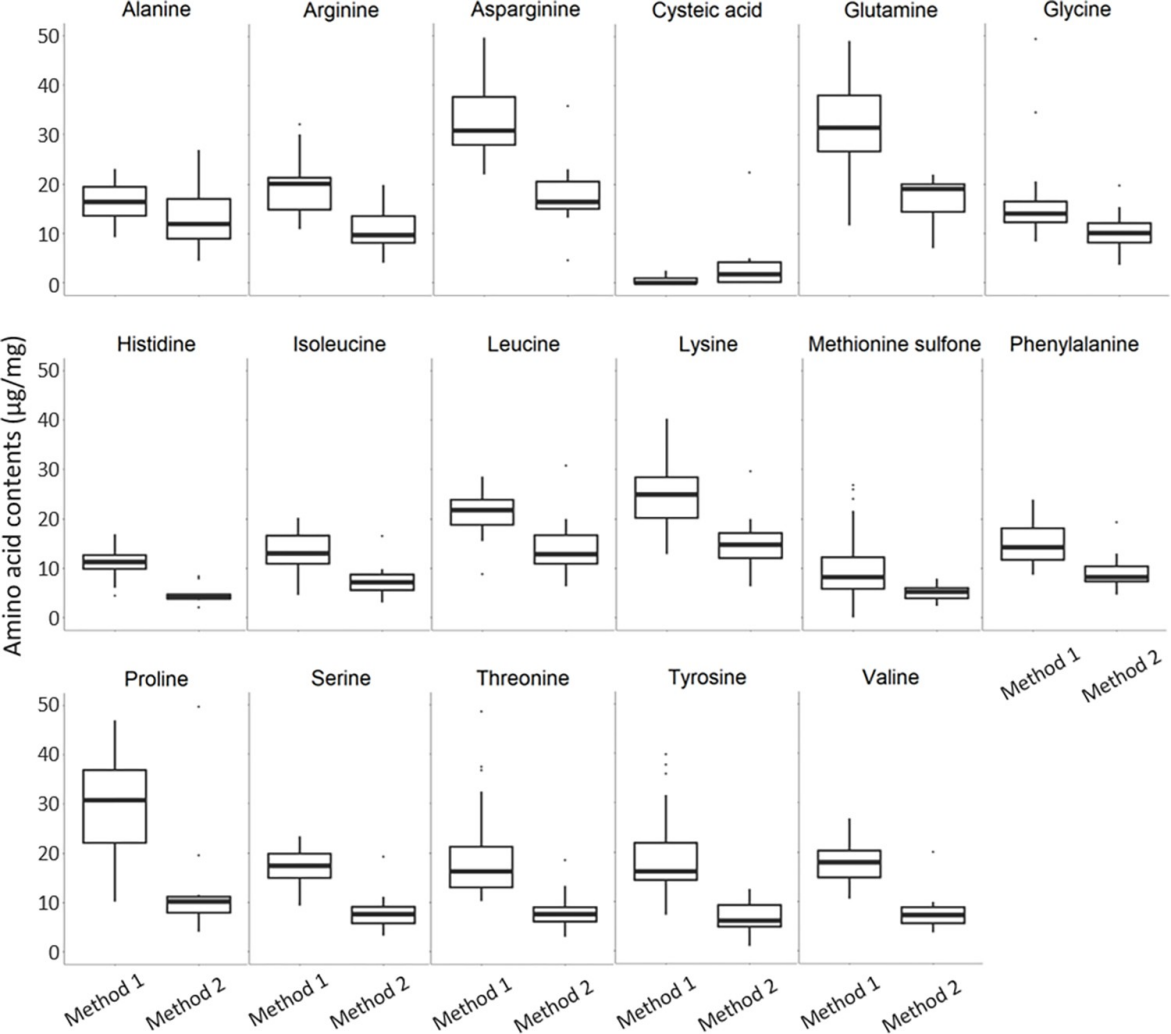
Boxplots of the contents of each amino acid (μg/mg) according to the IEX chromatography method (Method 1) and the HPLC method (Method 2).

Except for cysteine, species with high amino acid content were discriminated by the nMDS 1 axis from species with low amino acid content. Pollen that was particularly rich in cysteine was discriminated by the nMDS 2 axis from pollen that was particularly rich in glutamine, isoleucine, and leucine (Fig 2). High amino acid contents were linked to species analyzed with the IEX chromatography method, except for cysteine (Fig 3).

The amino acid content was significantly influenced by the method used, the plant family, and the interaction between these two factors (S2 Table). Proline content from Fabaceae pollen was significantly higher using IEX chromatography than HPLC (*p*. *adj*. < 0.05). Pollen from Lamiaceae had a significantly higher threonine content using IEX chromatography than HPLC (*p*. *adj*. < 0.01), as well as pollen from Boraginaceae for both threonine and tyrosine contents (*p*. *adj*. < 0.01), and pollen from Fabaceae (*p*. *adj*. < 0.05) and Lamiaceae (*p*. *adj*. < 0.05) for tyrosine content. Contents of glycine, arginine, histidine, and lysine were significantly dependent on the ‘family’ factor. Moreover, contents of proline (a non-essential amino acid), threonine, and tyrosine (essential amino acids) were significantly influenced by the interaction between ‘family’ and ‘method’ factors.

#### Amino acid scores

The AAS significantly differed among the 32 species according to the plant family (*F* = 2.784, *d*.*f*. = 6, *p* < 0.05), but not according to the method used. Pollen from 19 out of 32 species analysed had the maximum amino acid score (AAS = 1), including several species belonging to the Rosaceae, Lamiaceae, and Sapindaceae families, whereas 12 species were deficient in one or two essential amino acids (S1 Table). In other words, a species with a score of 1 has all the essential amino acids, without any deficiency, whereas a lower score indicates that there is a deficiency in one or several essential amino acids. The most common limiting essential amino acids were isoleucine (9 species) and methionine (4 species). Pollen of the Boraginaceae species had a low AAS (0.14–0.64), as did pollen from Fabaceae species like *Robinia pseudoacacia* (0.04), *Trifolium pratense* (0.29), and *Lotus corniculatus* (0.55), and the Boraginaceae species *Borago officinalis* (0.44), *Symphytum officinale* (0.43), and *Echium vulgare* (0.52, S1 Table).

## Discussion

### Interspecific differences in amino acid profiles

Amino acid profiles and contents differed among the studied plant species. At the family level, differences in total amino acid contents among plant families were higher than within each plant family. The interaction between ‘method’ and ‘family’ was not significant, indicating a similar effect of the analytical method on total amino acid contents regardless of the plant family. Our results suggested a phylogenetic influence on total amino acid contents that needs further testing with additional plant species. AASs also significantly differed among plant species. Pollen from the Rosaceae, Lamiaceae, and Sapindaceae species had high AASs unlike pollen from the Fabaceae and Boraginaceae families, which had deficiencies in one or several essential amino acids. These results are surprising since the majority of species in these families are considered highly attractive to bees [17, 46]. In the Fabaceae, *Medicago sativa* and *Cytisus scoparius* pollen contained the required essential amino acid contents, whereas other attractive bee-pollinated species like clovers (*Trifolium pratense* and *T*. *repens*) had deficiencies in amino acid composition (AAS = 0.29 and 0.56, respectively). Additionally, species belonging to the Rosaceae, whose open flowers are also intensively visited by bees, had low total amino acid contents. A low AAS does not in itself constitute a problem if the insect’s diet is supplemented with other pollen sources with high concentrations of the deficient amino acids. This could explain why an individual bee, to balance its diet, collects pollen from different floral sources during the same foraging bout.

To conserve energy and increase efficiency, bees will most often visit plant species that produce lots of floral resources (nectar and/or pollen), or produce many flowers at the same time. As also hypothesized in other studies, we posit that these species might produce large quantities of low-quality pollen, instead of low quantities of high-quality pollen [12, 24, 36, 37]. For example, several bee-pollinated species, like Asteraceae, provide a valuable source of nectar, whereas their pollen is deficient in certain nutrients [12, 36, 47]. Moreover, the pollen directly consumed or accumulated in bee baskets is lost for pollination. To prevent pollen consumption, several species offer toxic pollen, or protect their pollen from pollen eaters by producing secondary metabolites such as alkaloids, saponins, and/or terpenoids in the pollen [36, 48].

Amino acid composition, i.e., contents of essential (arginine, histidine, lysine, and threonine) and non-essential amino acids (glycine, proline, and tyrosine) differed among plant families. A possible synergistic biosynthesis pathway between several amino acids contents should be investigated. Proline, for example, is required for egg-laying by the queen [49] and high-proline pollen is preferred by the bees [50]. Proline is also used as fuel for bee flight because it is rapidly metabolized [51].

Three amino acids with the highest concentrations (asparagine, glutamine, and proline, S1 Table) among the pollen of the 32 species studied are not considered essential amino acids [26] whereas leucine and lysine (among the 5 most abundant amino acids in our samples) are essential. Our results are consistent with previous studies indicating that the six most common amino acids (arginine, asparagine, glutamine, leucine, lysine, and proline) constitute about 60% of the pollen protein content [52, 53]. Bees usually prefer pollen with high concentrations of essential amino acids, particularly isoleucine, leucine, and valine [54]. Nevertheless, little is known about the nutrient requirements of wild bees. For example, if all amino acids considered essential for honeybees are required in similar proportions or if non-essential amino acids like aspartic acid, glutamic acid, and proline can be used as energy and nitrogen sources remain unclear [51].

### Influence of the chromatography method

Amino acid profiles were quantified with two different analytical methods which significantly influenced both the composition and the total amino acid content. Except for cysteine, all amino acids were detected by the chromatography methods in a similar pattern, with a higher total amino acid content with IEX chromatography compared to HPLC.

The two chromatography methods included a two-step analysis involving hydrolysis under different conditions (acidic *versus* oxidative basic), which influenced the extraction of several amino acids. The combination of the amino acid quantification results after the two hydrolyses provided a global view of the amino acid profiles. For the IEX chromatography method, the complete analysis (including the two hydrolyses) was replicated several times (n = 3–6) for a better homogenization. By contrast, for the HPLC method, two replicas were devoted to the procedure with acid hydrolysis only and one to the procedure under oxidative conditions.

The IEX and HPLC methods differed in several ways during the amino acid detection process. The time required for acid hydrolysis with the IEX protocol was 24 hours, whereas the HPLC protocol required only 18 hours, which could explain the lower amino acid levels detected, if hydrolysis was not complete. Furthermore, the pH of the buffer injected in the column was acidic (2.2) in the IEX chromatography method whereas the pH was basic (10.4) in the HPLC method; therefore, the detection of the amino acids would be influenced by their protonated or non-protonated form. The higher content detected with HPLC only for cysteine could be explained by the addition of mercaptoethanol to the HCl solution [55]. As most amino acids are not detectable by absorption or fluorescence, they have to be derivatised to be detected. Amino acids are detected and quantified according to the intensity of the light emitted by the chromophores; therefore, the differences in the derivatization agents used in the two methods may explain the differences in chromophores produced and, consequently, their signal intensity detection.

IEX chromatography followed the more time-consuming Steine and Moore protocol compared with the HPLC system but is considered to be more reliable. Specific amino acid analysers (Biochrome) allow automation of the detection of amino acids and higher detection and separation of amino acids than a standard HPLC apparatus [56]. Therefore, for better comparability between studies, we strongly recommend using identical methods with similar hydrolysis and detection standards (with a specific amino acid detector) for future analyses of pollen chemistry.

## Conclusion

For future pollen chemical analyses, having a typical amino acid reference profile for congeneric or related species (from the same family) would enable prediction of the amino acid quantities available in plant pollen at a given time and place. Our results suggested a phylogenetic determinant for pollen total amino acid contents and profiles, as already detected for crude proteins and protein/lipid ratios, and for pollen sterols. Data on more species are needed to confirm such phylogenetic signals. IEX chromatography is preferred for further studies because, although more time consuming, the results obtained are more robust, consistent, and repeatable than with standard HPLC chromatography. Detection of evolutionary or ecological trends needs standardized sampling, protocols, and chemical analyses. Chemical analyses can influence the total and the amino acid composition. Thus, analysis methods should be based on similar hydrolysis and detection standards (with a specific amino acid detector).

For future pollinator conservation programs, precise pollen chemical composition analyses will help in determining the floral species best-suited for pollinator conservation plantations to ensure diverse and high-quality pollen resources to balance pollinator diet and promote pollinator health.

## Supporting information

S1 Fig Boxplot of total amino acids according to the analytical method used. (A) Boxplot with the 32 species studied. (B) Boxplot with 6 species analysed by the two methods. Method 1: IEX chromatography method; Method 2: HPLC method.
(TIF)

S1 Table Contents of each amino acid and total amino acids (μg/mg) for the 32 studied species. Pollen chemical analysis method (1: IEX chromatography method; 2: HPLC method) and data sources are indicated (1: Somme et al. 2016 [39]; 2: Moquet et al. 2016 [57]; 3: Quinet et al. 2016 [58]; 4: Roger et al. 2017 [14]; 5: Carlier 2020 [31]). Amino acid score (AAS), most limiting amino acids (iso, isoleucine; met, methionine; val, valine) for the 32 studied species. Plant names follow APGIV classification [42]. The amino acids in bold are considered essential amino acids for honeybees [26]. (A, Asteraceae; B, Boraginaceae; F, Fabaceae; L, Lamiaceae; M, Malvaceae; R, Rosaceae; S, Sapindaceae).
(DOCX)

S2 Table MANOVA table reporting the results of a multivariate comparison evaluating the amino acids contents according to method, family, and the interaction between these two factors. Repeated measures ANOVA table for univariate comparisons of each amino acid content. Significant p-values are noted in bold.
(DOCX)
