# Supplementary figures and images for: Some bee-pollinated plants provide nutritionally incomplete pollen amino acid resources to their pollinators
Source: PLoS One. 2022 Aug 2;17(8):e0269992. doi: 10.1371/journal.pone.0269992 (PMC9345472; doi:10.1371/journal.pone.0269992)

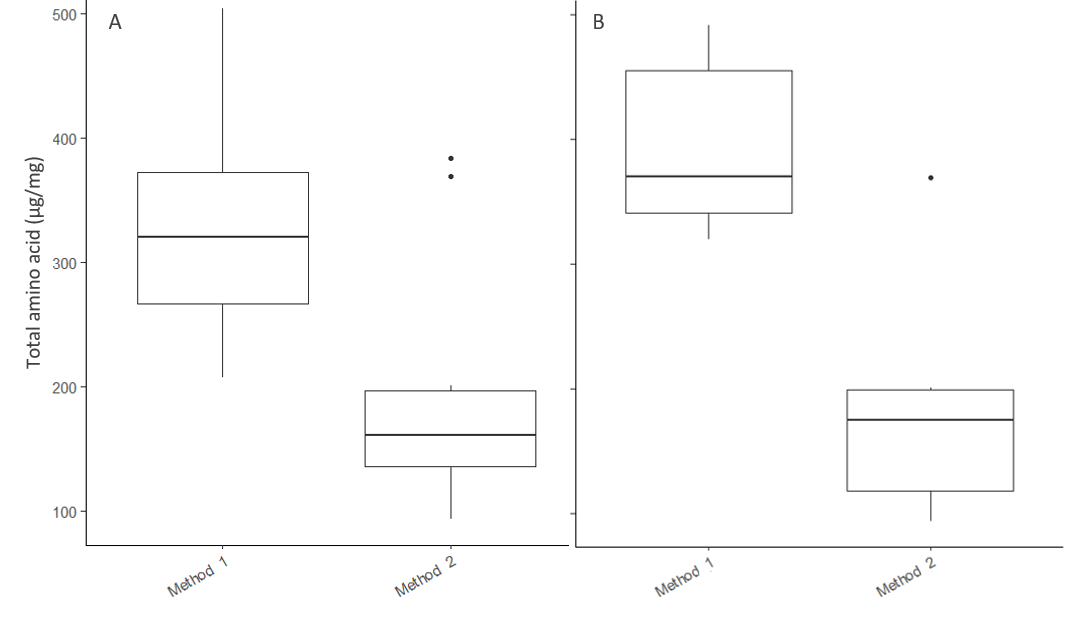

Supplement: S1 Fig — (A) Boxplot with the 32 species studied. (B) Boxplot with 6 species analysed by the two methods. Method 1: IEX chromatography method; Method 2: HPLC method. (TIF) [file pone.0269992.s001.tif]
