# Supplementary material for: Some bee-pollinated plants provide nutritionally incomplete pollen amino acid resources to their pollinators
Source: PLoS One. 2022 Aug 2;17(8):e0269992. doi: 10.1371/journal.pone.0269992 (PMC9345472; doi:10.1371/journal.pone.0269992)
Supplement: S1 Table — Pollen chemical analysis method (1: IEX chromatography method; 2: HPLC method) and data sources are indicated (1: Somme et al. 2016 [39]; 2: Moquet et al. 2016 [57]; 3: Quinet et al. 2016 [58]; 4: Roger et al. 2017 [14]; 5: Carlier 2020 [31]). Amino acid score (AAS), most limiting amino acids (iso, isoleucine; met, methionine; val, valine) for the 32 studied species. Plant names follow APGIV classification [42]. The amino acids in bold are considered essential amino acids for honeybees [26]. (A, Asteraceae; B, Boraginaceae; F, Fabaceae; L, Lamiaceae; M, Malvaceae; R, Rosaceae; S, Sapindaceae). (DOCX) [file pone.0269992.s002.docx]

**S1 Table. Contents of each amino acid and total amino acids (µg/mg) for the 32 studied species.** Pollen chemical analysis method (1: IEX chromatography method; 2: HPLC method) and data sources are indicated (1: Somme et al. 2016 [39]; 2: Moquet et al. 2016 [58]; 3: Quinet et al. 2016 [59]; 4: Roger et al. 2017 [14]; 5: Carlier 2020 [31]). Amino acid score (AAS), most limiting amino acids (iso, isoleucine; met, methionine; val, valine) for the 32 studied species. Plant names follow APGIV classification [43]. The amino acids in bold are considered essential amino acids for honeybees [26]. (A, Asteraceae; B, Boraginaceae; F, Fabaceae; L, Lamiaceae; M, Malvaceae; R, Rosaceae; S, Sapindaceae)

| Plant species | | Plant family | Pollen collection technique | Method | Data sources | Alanine | Arginine | | Asparginine | | Cysteine | | Glutamine | | Glycine | | Histidine | | Isoleucine | | Leucine | | Lysine | | Methionine | | Phenylalanine | | Proline | | Serine | | Threonine | | Tyrosine | | Valine | | Total amino acid | AAS | | Most limiting amino acid | |
| --- | --- | --- | --- | --- | --- | --- | --- | --- | --- | --- | --- | --- | --- | --- | --- | --- | --- | --- | --- | --- | --- | --- | --- | --- | --- | --- | --- | --- | --- | --- | --- | --- | --- | --- | --- | --- | --- | --- | --- | --- | --- | --- | --- |
| *Centaurea jacea* | | A | Anther | 1 | 2,4 | 19.65 | 14.70 | | 29.76 | | 0.00 | | 32.52 | | 16.99 | | 12.47 | | 11.58 | | 19.67 | | 24.57 | | 7.39 | | 11.51 | | 34.29 | | 17.70 | | 16.70 | | 15.88 | | 16.02 | | 301.41 | 0.94 | | Iso | |
| *Cyanus segetum* | | A | Anther | 1 | 2 | 22.29 | 15.60 | | 31.78 | | 0.00 | | 38.10 | | 16.15 | | 12.89 | | 13.51 | | 22.76 | | 26.13 | | 5.68 | | 11.65 | | 36.35 | | 19.97 | | 14.50 | | 15.31 | | 16.70 | | 319.37 | 1.00 | | 0 | |
| *Cyanus segetum* | | A | Anther | 2 | 5 | 22.18 | 15.79 | | 20.06 | | NA | | 22.01 | | 15.37 | | 7.97 | | 9.84 | | 19.93 | | 10.80 | | NA | | 12.94 | | 8.03 | | 11.14 | | 13.37 | | 10.94 | | NA | | 200.37 | NA | | NA | |
| *Borago officinalis* | | B | Anther | 2 | 5 | 4.37 | 11.19 | | 4.59 | | 0.77 | | 7.01 | | 10.76 | | 4.74 | | 3.07 | | 7.67 | | 16.19 | | 3.11 | | 4.65 | | 8.13 | | 4.51 | | 2.97 | | 0.98 | | 6.10 | | 369.44 | 0.14 | | Iso | |
| *Borago officinalis* | | B | Anther | 1 | 4 | 18.80 | 24.10 | | 49.60 | | 0.00 | | 24.90 | | 70.40 | | 14.20 | | 12.60 | | 21.50 | | 35.10 | | 24.10 | | 19.90 | | 47.00 | | 20.70 | | 48.70 | | 35.90 | | 24.00 | | 491.50 | 0.44 | | Iso | |
| *Echium vulgare* | | B | Anther | 1 | 4 | 19.80 | 23.10 | | 37.70 | | 1.00 | | 41.60 | | 15.90 | | 13.10 | | 20.20 | | 28.00 | | 27.40 | | 2.80 | | 20.50 | | 32.90 | | 19.10 | | 21.20 | | 17.20 | | 19.70 | | 361.20 | 0.52 | | Met | |
| *Symphytum officinale* | | B | Anther | 1 | 4 | 22.10 | 30.10 | | 53.60 | | 0.00 | | 27.20 | | 34.50 | | 15.90 | | 8.70 | | 23.90 | | 40.30 | | 26.90 | | 23.90 | | 54.90 | | 23.10 | | 52.60 | | 39.80 | | 26.50 | | 504.00 | 0.43 | | Iso | |
| *Cytisus scoparius* | | F | Anther | 2 | 5 | 12.50 | 6.36 | | 13.12 | | NA | | 10.19 | | 8.42 | | 3.52 | | 4.06 | | 9.20 | | 6.73 | | NA | | 6.68 | | 10.45 | | 4.66 | | 5.23 | | 4.84 | | NA | | 105.95 | NA | | NA | |
| *Cytisus scoparius* | | F | Anther | 1 | 1,2 | 18.29 | 24.23 | | 33.59 | | 1.42 | | 38.99 | | 14.35 | | 14.06 | | 19.57 | | 26.52 | | 26.80 | | 11.79 | | 21.34 | | 46.66 | | 18.38 | | 18.06 | | 18.59 | | 19.04 | | 371.68 | 1.00 | | 0 | |
| *Lotus corniculatus* | | F | Tiny | 1 | 4 | 15.10 | 21.40 | | 33.10 | | 0.00 | | 19.70 | | 49.40 | | 10.00 | | 8.20 | | 15.60 | | 26.60 | | 19.30 | | 17.00 | | 38.90 | | 16.30 | | 32.40 | | 28.50 | | 20.60 | | 372.10 | 0.55 | | Iso | |
| *Medicago sativa* | | F | Anther | 2 | 5 | 12.57 | 9.54 | | 21.91 | | 0.00 | | 19.12 | | 11.09 | | 4.71 | | 7.75 | | 15.05 | | 17.19 | | 5.63 | | 10.35 | | 10.22 | | 8.99 | | 8.54 | | 7.82 | | 7.28 | | 166.80 | 1.00 | | 0 | |
| *Robinia pseudoacacia* | | F | Anther | 1 | 1 | 22.99 | 21.27 | | 37.01 | | 0.00 | | 43.66 | | 16.31 | | 12.82 | | 16.81 | | 27.30 | | 25.96 | | 0.00 | | 12.10 | | 60.13 | | 21.09 | | 16.67 | | 20.55 | | 20.39 | | 375.08 | 0.04 | | Met | |
| *Trifolium pratense* | | F | Tiny | 1 | 4 | 15.30 | 20.80 | | 38.20 | | 0.00 | | 20.60 | | 57.60 | | 12.30 | | 4.70 | | 17.80 | | 30.60 | | 21.60 | | 13.20 | | 40.80 | | 19.70 | | 37.50 | | 30.70 | | 21.80 | | 403.20 | 0.29 | | Iso | |
| *Trifolium repens* | | F | Tiny | 1 | 4 | 16.30 | 20.70 | | 37.50 | | 0.00 | | 21.70 | | 58.40 | | 11.60 | | 9.30 | | 18.10 | | 30.50 | | 21.50 | | 17.90 | | 43.80 | | 17.40 | | 36.80 | | 31.50 | | 21.20 | | 414.20 | 0.54 | | Iso | |
| *Lamium album* | | L | Anther | 2 | 5 | 11.40 | 9.99 | | 15.32 | | 4.55 | | 19.46 | | 9.00 | | 4.00 | | 7.12 | | 13.51 | | 14.12 | | 4.04 | | 8.44 | | 11.09 | | 7.29 | | 7.75 | | 6.45 | | 7.37 | | 154.28 | 1.00 | | 0 | |
| *Lamium album* | | L | Anther | 1 | 4 | 22.30 | 32.20 | | 52.00 | | 0.00 | | 26.50 | | 18.40 | | 16.90 | | 11.50 | | 23.80 | | 35.30 | | 26.00 | | 21.90 | | 57.10 | | 23.40 | | 51.10 | | 37.70 | | 26.80 | | 482.90 | 0.40 | | Iso | |
| *Lamium galeobdolon* | | L | Anther | 2 | 5 | 16.77 | 13.67 | | 17.54 | | 2.00 | | 21.52 | | 12.01 | | 4.77 | | 8.60 | | 17.27 | | 19.98 | | 7.05 | | 10.45 | | 10.37 | | 9.43 | | 9.49 | | 10.08 | | 8.88 | | 190.12 | 1.00 | | 0 | |
| *Malva moschata* | | M | Fresh | 2 | 5 | 9.02 | 8.19 | | 14.38 | | 3.13 | | 19.37 | | 7.25 | | 3.96 | | 6.52 | | 11.57 | | 12.69 | | 5.25 | | 8.03 | | 7.88 | | 7.07 | | 7.49 | | 5.86 | | 5.18 | | 135.97 | 0.95 | | Val | |
| *Tilia cordata* | | M | Anther | 1 | 1,2 | 11.66 | 13.43 | | 26.60 | | 2.47 | | 28.53 | | 10.71 | | 9.13 | | 12.63 | | 18.72 | | 18.29 | | 6.83 | | 13.29 | | 22.12 | | 13.41 | | 11.69 | | 9.93 | | 13.67 | | 243.14 | 1.00 | | 0 | |
| *Tilia platyphyllos* | | M | Anther | 1 | 1 | 18.60 | 15.64 | | 43.97 | | 0.00 | | 43.57 | | 16.63 | | 10.77 | | 17.20 | | 25.24 | | 24.05 | | 0.00 | | 9.42 | | 24.59 | | 20.40 | | 15.88 | | 18.00 | | 17.49 | | 321.44 | 0.00 | | Met | |
| *Tilia tomentosa* | | M | Anther | 1 | 1 | 10.75 | 10.91 | | 25.34 | | 1.56 | | 24.48 | | 9.99 | | 8.24 | | 10.84 | | 15.67 | | 15.66 | | 5.61 | | 10.76 | | 16.63 | | 12.19 | | 10.24 | | 8.05 | | 12.40 | | 209.33 | 1.00 | | 0 | |
| *Tilia x euchlora* | | M | Anther | 1 | 1 | 11.17 | 14.54 | | 26.41 | | 0.53 | | 28.24 | | 11.12 | | 10.10 | | 14.77 | | 19.18 | | 18.66 | | 7.52 | | 14.42 | | 16.03 | | 13.72 | | 12.31 | | 10.86 | | 14.29 | | 243.89 | 1.00 | | 0 | |
| *Tilia x vulgaris* | | M | Anther | 1 | 1 | 13.25 | 13.81 | | 28.34 | | 2.18 | | 29.60 | | 12.52 | | 11.05 | | 15.59 | | 19.65 | | 18.83 | | 7.37 | | 14.27 | | 21.68 | | 14.79 | | 12.41 | | 10.35 | | 14.89 | | 260.59 | 1.00 | | 0 | |
| *Crataegus laevigata* | | R | Anther | 2 | 5 | 26.91 | 19.90 | | 35.80 | | 22.41 | | 52.56 | | 19.74 | | 8.42 | | 16.56 | | 30.79 | | 29.65 | | 7.92 | | 19.35 | | 49.78 | | 19.24 | | 18.55 | | 12.56 | | 20.08 | | 383.36 | 1.00 | | 0 | |
| *Filipendula ulmaria* | | R | Anther | 1 | 2 | 14.70 | 19.11 | | 29.53 | | 0.00 | | 38.17 | | 12.00 | | 8.97 | | 12.01 | | 21.36 | | 24.50 | | 6.53 | | 9.28 | | 30.80 | | 15.94 | | 13.22 | | 14.80 | | 14.89 | | 284.17 | 1.00 | | 0 | |
| *Fragaria vesca* | | R | Tiny | 2 | 5 | 9.78 | 8.02 | | 19.69 | | 1.63 | | 16.49 | | 7.23 | | 4.02 | | 5.97 | | 12.01 | | 12.59 | | 4.01 | | 7.67 | | 11.59 | | 6.20 | | 6.46 | | 5.64 | | 5.63 | | 137.52 | 1.00 | | 0 | |
| *Malus domestica* | | R | Anther | 1 | 2, 3 | 12.35 | 11.03 | | 54.74 | | 0.00 | | 31.11 | | 8.32 | | 4.52 | | 8.80 | | 15.47 | | 12.90 | | 2.57 | | 11.77 | | 10.21 | | 11.65 | | 10.52 | | 7.30 | | 10.49 | | 223.58 | 0.77 | | Met | |
| *Prunus avium* | | R | Anther | 2 | 5 | 8.74 | 8.38 | | 15.18 | | 0.00 | | 20.71 | | 9.40 | | 3.80 | | 7.25 | | 12.19 | | 15.57 | | NA | | 7.81 | | 4.76 | | 8.08 | | 7.44 | | 4.96 | | NA | | 134.27 | NA | | NA | |
| *Prunus cerasus* | | R | Anther | 1 | 4 | 9.20 | 13.30 | | 21.90 | | 0.00 | | 11.70 | | 13.80 | | 6.10 | | 4.60 | | 8.90 | | 16.00 | | 11.40 | | 8.70 | | 24.30 | | 9.30 | | 21.50 | | 14.70 | | 11.40 | | 206.80 | 0.54 | | Iso | |
| *Pyrus communis* | | R | Anther | 2 | 5 | 4.39 | 4.09 | | 22.98 | | 0.00 | | 12.43 | | 3.65 | | 2.15 | | 4.57 | | 6.32 | | 6.31 | | 2.42 | | 5.28 | | 4.04 | | 3.28 | | 4.39 | | 2.98 | | 3.72 | | 92.98 | 1.00 | | 0 | |
| *Pyrus communis* | | R | Anther | 1 | 3 | 20.61 | 21.24 | | 49.65 | | 0.00 | | 49.11 | | 15.28 | | 11.38 | | 16.96 | | 28.54 | | 28.78 | | 4.20 | | 13.87 | | 30.24 | | 21.19 | | 17.32 | | 20.38 | | 20.31 | | 368.69 | 0.74 | | Met | |
| *Rubus aggr.* | | R | Anther | 1 | 2 | 17.47 | 20.05 | | 44.40 | | 0.00 | | 36.31 | | 20.54 | | 9.92 | | 12.76 | | 23.10 | | 29.06 | | 12.45 | | 11.70 | | 37.47 | | 18.25 | | 26.09 | | 22.35 | | 19.48 | | 361.38 | 0.88 | | Iso | |
| *Sorbus aucuparia* | | R | Anther | 1 | 2 | 16.38 | 19.96 | | 30.07 | | 0.53 | | 35.14 | | 12.67 | | 11.79 | | 16.53 | | 23.34 | | 25.17 | | 10.70 | | 17.96 | | 32.00 | | 16.95 | | 15.96 | | 14.88 | | 17.88 | | 317.91 | 1.00 | | 0 | |
| *Acer platanoides* | | S | Tiny | 1 | 2 | 12.45 | 17.11 | | 22.52 | | 0.99 | | 27.23 | | 10.82 | | 8.23 | | 13.35 | | 19.51 | | 19.32 | | 6.99 | | 14.31 | | 11.42 | | 13.76 | | 12.76 | | 11.54 | | 14.76 | | 237.04 | 1.00 | | 0 | |
| *Acer pseudoplatanus* | | S | Tiny | 1 | 1, 2 | 14.43 | 20.36 | | 27.58 | | 2.19 | | 31.94 | | 12.47 | | 10.50 | | 15.49 | | 22.19 | | 22.88 | | 9.05 | | 16.89 | | 22.28 | | 15.74 | | 15.01 | | 14.32 | | 16.89 | | 290.21 | 1.00 | | 0 | |
| *Aesculus carnea* | | S | Anther | 1 | 1 | 16.44 | 20.19 | | 28.62 | | 1.55 | | 36.13 | | 13.06 | | 11.23 | | 16.71 | | 23.93 | | 23.59 | | 10.48 | | 18.20 | | 32.35 | | 16.61 | | 15.93 | | 15.19 | | 18.00 | | 318.22 | 1.00 | | 0 | |
| *Aesculus hippocastanum* | | S | Anther | 1 | 1 | 16.87 | 22.96 | | 30.79 | | 0.90 | | 37.87 | | 14.01 | | 12.30 | | 17.64 | | 24.91 | | 24.80 | | 11.42 | | 19.17 | | 28.94 | | 17.39 | | 16.94 | | 16.40 | | 18.41 | | 331.72 | 1.00 | | 0 | |
| *Aesculus hippocastanum* | | S | Anther | 2 | 5 | 17.57 | 13.58 | | 15.16 | | 5.03 | | 18.61 | | 12.46 | | 4.53 | | 9.29 | | 16.55 | | 17.13 | | 6.11 | | 10.42 | | 19.65 | | 8.27 | | 8.92 | | 9.06 | | 9.93 | | 195.51 | 1.00 | | 0 | |
|  | Mean ± SD amino acids with method 1 | | | | | 16.51 ± 3.93 | | 19.30 ± 5.26 | | 35.55 ± 9.92 | | 0.59 ± 0.82 | | 31.72 ± 8.65 | | 21.63 ± 17.20 | | 11.17 ± 2.74 | | 13.18 ± 4.14 | | 21.33 ± 4.53 | | 25.07 ± 6.41 | | 10.78 ± 7.80 | | 15.19 ± 4.27 | | 32.84 ± 13.42 | | 17.24 ± 3.55 | | 22.08 ± 12.87 | | 19.26 ± 9.22 | | 18.00 ± 4.17 | 331.34 ± 83.81 | |  | |  |
|  | Mean ± SD amino acids with method 2 | | | | | 12.93 ± 6.80 | | 11.20 ± 4.38 | | 19.68 ± 7.41 | | 3.51 ± 6.74 | | 21.05 ± 11.30 | | 11.30 ± 4.17 | | 5.27 ± 1.78 | | 7.87 ± 3.51 | | 14.74 ± 6.53 | | 15.57 ± 6.21 | | 5.58 ± 1.82 | | 9.74 ± 3.93 | | 14.10 ± 12.23 | | 8.93 ± 4.15 | | 9.48 ± 4.17 | | 7.70 ± 3.37 | | 9.14 ± 4.83 | 200.20 ± 93.80 | |  | |  |
