# Supplementary material for: Some bee-pollinated plants provide nutritionally incomplete pollen amino acid resources to their pollinators
Source: PLoS One. 2022 Aug 2;17(8):e0269992. doi: 10.1371/journal.pone.0269992 (PMC9345472; doi:10.1371/journal.pone.0269992)
Supplement: S2 Table — Repeated measures ANOVA table for univariate comparisons of each amino acid content. Significant p-values are noted in bold. (DOCX) [file pone.0269992.s003.docx]

**S2 Table.** **MANOVA table reporting the results of a multivariate comparison evaluating the amino acids contents according to method, family, and the interaction between these two factors. Repeated measures ANOVA table for univariate comparisons of each amino acid content.** **Significant p-values are noted in bold.**

| **Repeated Measures ANOVA** | **Method** | | **Family** | | **Method * Family** | |
| --- | --- | --- | --- | --- | --- | --- |
|  | *F value* | *P value* | *F value* | *P value* | *F value* | *P value* |
| Response |  |  |  |  |  |  |
| Alanine | 4.45 | **0.04** | 1.04 | 0.37 | 1.52 | 0.22 |
| Arginine | 34.76 | **6.22E-03** | 5.13 | **0.002** | 2.04 | 0.11 |
| Aspartic acid | 29.38 | **1.92E-02** | 2.13 | 0.09 | 2.29 | 0.08 |
| Cysteine | 6.57 | **0.02** | 0.28 | 0.94 | 0.82 | 0.55 |
| Glutamine | 6.52 | **0.02** | 0.33 | 0.91 | 0.33 | 0.89 |
| Glycine | 4.98 | **0.04** | 3 | **0.03** | 0.87 | 0.52 |
| Histidine | 70.01 | **2.78E-05** | 2.93 | **0.03** | 2.22 | 0.09 |
| Isoleucine | 9.56 | **0.005** | 0.36 | 0.9 | 0.41 | 0.83 |
| Leucine | 8.7 | **0.007** | 0.19 | 0.98 | 0.54 | 0.74 |
| Lysine | 16.59 | **0.0005** | 2.68 | **0.04** | 0.98 | 0.49 |
| Methionine | 5.7 | **0.03** | 1.98 | 0.11 | 1.3 | 0.30 |
| Phenylalanine | 18.16 | **0.0003** | 2.36 | 0.07 | 2.56 | 0.057 |
| Proline | 19.31 | **0.0002** | 2.92 | **0.03** | 2.72 | **0.05** |
| Serine | 37.61 | **3.58E-03** | 0.93 | 0.49 | 1.21 | 0.34 |
| Threonine | 21.42 | **0.0001** | 5.16 | **0.002** | 3.88 | **0.01** |
| Tyrosine | 32.43 | **9.97E-03** | 5.15 | **0.002** | 3.4 | **0.02** |
| Valine | 46.98 | **6.95E-04** | 2.28 | 0.07 | 2.035 | 0.11 |
